# Schizophrenia and quality of life: a one-year follow-up in four EU countries

**DOI:** 10.1186/1471-244X-6-39

**Published:** 2006-09-19

**Authors:** Viviane Kovess-Masféty, Miguel Xavier, Berta Moreno Kustner, Agnieszka Suchocka, Christine Sevilla-Dedieu, Jacques Dubuis, Elisabeth Lacalmontie, Jacques Pellet, Jean-Luc Roelandt, Dermot Walsh

**Affiliations:** 1 MGEN Foundation for Public Health, EA 4069 University of Paris 5, 3, square Max Hymans, 75748 Paris Cedex 15, France; 2 Clinica Universitaria de Psiquiatria e Saude Mental, Faculdade de Ciências Medicas, calçada da Tapada, 155, 1300 Lisboa, Portugal; 3 Departamento de Psiquiatria, Facultad de Medicina, avenida de Madrid, s/n, 18071 Granada, Spain; 4 CHS Le Vinatier; 95, boulevard Pinel, 69677 Bron Cedex, France; 5 CHS La Verrière, 78321 Le Mesnil St Denis, France; 6 Service Universitaire de Psychiatrie Adultes, CHU St Etienne, 42055 Saint Etienne Cedex 02, France; 7 Clinique Jérôme Bosch, 104, rue du Général Leclerc, BP 10, 59487 Armentières Cedex, France; 8 Health Research Board, Holbrook House, Holles Street, Dublin 2, Ireland

## Abstract

**Background:**

This article systematically monitors the quality of life (QOL) of patients with schizophrenia from seven different sites across four European countries: France, Ireland, Portugal and Spain.

**Methods:**

A one-year prospective cohort study was carried out. Inclusion criteria for patients were: a clinical lifetime diagnosis of schizophrenia according to ICD-10 (F20) diagnostic criteria for research, age between 18 and 65 years and at least one contact with mental health services in 1993. Data concerning QOL were recorded in seven sites from four countries: France, Portugal, Ireland and Spain, and were obtained using the Baker and Intagliata scale. At baseline, 339 patients answered the QOL questionnaire. At one-year follow-up, Spain could not participate, so only 263 patients were contacted and 219 agreed to take part. QOL was compared across centres by areas and according to a global index. QOL was correlated with presence of clinical and social problems, needs for care and interventions provided during the one-year follow-up.

**Results:**

We did not find any link between gender and QOL. There were some significant differences between centres concerning many items. What is more, these differences were relative: in Lisbon where the lowest level of satisfaction was recorded, people were satisfied with food but highly dissatisfied with finances, whereas in St Etienne, where the highest level of satisfaction was recorded, people were less satisfied with food when they were more satisfied with finances. The evolution in one year among those respondents who took part in the follow-up (excluding the subjects from Granada) showed different patterns depending on the items.

**Conclusion:**

The four countries have different resources and patients live in rather different conditions. However, the main differences as far as their QOL is concerned very much depend on extra-psychiatric variables, principally marital status and income.

## Background

This article compares the quality of life of patients with schizophrenia in seven centres in four European countries: France (with centres in Lille, Lyon, La Verrière and St Etienne), Ireland (Dublin), Portugal (Lisbon) and Spain (Granada), and relates this to social and clinical characteristics of patients within their different psychiatric systems.

Indeed, there are large differences between these countries, mainly because of diverse historical backgrounds and the different resources made available for the care of patients with severe mental illness. In addition, the deinstitutionalization process has been implemented at various levels over the last twelve years in most European countries. Consequently, the ratio of psychiatric beds per 1,000 inhabitants remains high in some countries, whereas in others this ratio is low, either because of an effort to decrease it or because of a lack of availability. Moreover, alternatives to long-term hospitalisation such as sheltered housing are diversely developed, and in some countries the absence of such resources forces patients to live in their family's homes. The relationships between in-patient and out-patient care systems and the relationships between the psychiatric system and the primary care system vary substantially. As a result, continuity of care is ensured in varying degrees. It is also worth mentioning that social benefits for people suffering from severe psychiatric disorders vary greatly between countries, and are even totally lacking in some of them.

The broad impact of this diversity on the lives of the severely and persistently mentally ill and the resulting completion of the needs generated by such illnesses pose a particular challenge in the assessment of services for these persons [1]. Relevant outcome areas include psychiatric symptoms, functional status, and access to resources and opportunities, subjective well-being, burden to the family and community safety.

Because of this broad array of relevant outcomes and because of a prevailing concern that outcome assessments should include the patient's perspective, increased attention has been paid over the past decade to the development of patient "quality of life" measurements [2,3]. Assessment of the quality of life (QOL) is therefore emerging as an important criterion of the performance of services [4-6]. QOL approaches occupy, in a sense, an intermediate position between expert-defined assessments of need and client/user-defined demand. They are planned and used by experts, but often collect information about what the respondents value, want and prefer. QOL has become a valued assessment in those branches of medicine dealing with chronic suffering and disability [2]. QOL has also been largely applied to mental health patients since it embodies concern for patients as people and not just as cases [3]. In particular, QOL has been highly documented for patients with schizophrenia [7-16].

A systematic comparison and evaluation of the QOL of patients and their one-year evolution across some European sites will then bring useful information for planning purpose.

## Methods

### Sample

The present paper is based on a subset study on QOL conducted under the umbrella of the *European Research Group On Schizophrenia *(ERGOS).

In the main study, the patients came from a network of researchers and clinicians in 10 centres, from seven countries: France (with centres in Lille, Lyon, La Verrière and St Etienne), Germany (Mannheim), Ireland (Dublin), the Netherlands (Groningen), Portugal (Lisbon) and Spain (Granada). The study aimed to describe and compare the psychiatric care for a group of patients with chronic schizophrenia in a circumscribed geographical area in each participating country from Southern, Central and Northern Europe [17]. This one-year prospective cohort study included patients with a clinical lifetime diagnosis of schizophrenia according to ICD-10 (F20) diagnostic criteria for research [18], aged between 18 and 65 years old and who had at least one contact with mental health services during the year before inclusion. The selection of patients was conducted on the basis of the clinical diagnosis, which had to be confirmed by the use of a standardized interview schedule for present state: the *Schedules for Clinical Assessment in Neuropsychiatry *(SCAN) version 1.0 [19], which allowed for an assessment of lifetime representative episodes of schizophrenia. Patients were eligible for the study independently of whether they were receiving in- or out-patient care.

The results presented here concern a subset of seven centres from four countries: France, Portugal, Ireland and Spain, which agreed to collect additional data on QOL. As for the main study, the baseline assessment took place as soon as possible after a randomised selection from the list of eligible patients (those who had a lifelong diagnostic of schizophrenia assessed by SCAN) and after written informed consent following ethical committee recommendations. Finally, 419 patients constituted the target population (34 initially selected were not contacted because the required number was completed in that centre). 38 (9.0%) could not be contacted any more after selection, and 45 (10.7%) refused to participate in the study. Then, another 29, who participated in the study, did not complete the QOL assessment. So, the final QOL assessment concerned 339 patients at baseline (80.9% participation rate). At one-year follow-up, Spain could not participate in the QOL assessment, so the remaining eligible patients were 263 and only 219 agreed to take part (83.3% of the baseline population).

### Instruments

There are many QOL measures, which can be divided into generic and non generic. Since severe disorders are concerned we thought that a specific measure was needed and we selected the *Satisfaction with Life Domains Scale *(SLDS) [20,21]. This scale has the advantage of being one of the shortest QOL measures covering a variety of areas whilst allowing for a global score.

The SLDS was initially developed to evaluate the impact of the Community Support Program in New York State on the QOL of chronically mentally ill patients. It is a self-report scale administered by a trained interviewer and takes approximately 10 minutes to administer. Its individual items cover 15 areas of everyday life: satisfaction with housing, neighbourhood, food, clothing, health, people lived with, friends, family, relations with other people, work/day schedule, spare time, leisure time, services and facilities in the area, economic situation, and place lived in now compared with state hospital. Respondents chose for each area one face from among seven proposed, corresponding to different emotional states. The faces varied from very satisfied and happy to not satisfied at all and sad. These can be summed into a total life satisfaction score.

The SLDS has been extensively used with severe psychiatric patients in Belgium, Quebec and France. The French translation was available to us through a validation study conducted in Quebec [22]. This study allowed ensuring test-retest reliability, internal consistencies and discriminating power when translated in another language. On the other hand, the Spanish and Portuguese translations were done by bilingual experts from this field.

It has to be noted that the initial 15-item version of the SLDS was modified by C. Mercier and P. Corten who deleted 1 item (i.e., place lived in now compared with state hospital) and added 6 items covering love life, freedom and empowerment aspects. This modified version (20 items) was used by all centres except the Irish ones, which used the original 15-item version. Consequently, all results will be presented on the 14 common items for all centres, plus on the 6 added questions except for Dublin.

For the patients who did not answer some of the items of the QOL questionnaire, we attributed the value 4 (medium value) to the items in question in order to be able to compute the global score. Another solution would have been to use the mean or median value found for each item, however significance tests revealed that results would not have differed. For individual item comparisons, we are producing the number of patients by item.

The information concerning sociodemographic variables was collected by the *Past History and Sociodemographic Description schedule *(PHSD) [23], which provides information on the level of education, occupational situation, where the patient lives and information on his/her family. Given the variability in educational systems and standards of living across European countries, we had to slightly modify this instrument in order to harmonise the response scales for educational level, professional training and level of income (level of income and existence of a mandatory minimal wage, housing, social benefit based or regular income, variation in the level of attribution of social benefits for patients with schizophrenia according to the different national regulations).

The presence of a significant problem in various clinical and social domains was assessed at entry and followed up through the *Needs For Care Assessment Schedule *(NFCAS), a standardised procedure created by Brewin [24,25], which was designed to improve care planning for such patients and has been extensively used in various circumstances [26-28]. A specific inter-rater reliability study was set up to ensure that the standardised training procedures succeeded in obtaining an acceptable level of comparability across countries [29].

The interview also covered 11 areas of clinical functioning (psychotic symptoms, negative symptoms, side effects of medication, neurotic symptoms, dementia, physical problems, dangerous behaviour, socially embarrassing behaviour, distress, alcohol and drug use) and 11 areas of social functioning (personal hygiene, shopping, getting meals, household chores, use of public transport, use of public amenities, education, occupation, communication, finances and management of own affairs).

### Data analysis

The Chi-square test, or the Fisher-exact test, whenever the number of cases was below 5, was used to compare ratios and the Student or Fisher test was used to compare means. In addition, multiple linear regression was used to measure the effect of variables on the level of global satisfaction. The software was SPSS V11 and Epi Info V6. Statistical significance was set at 0.05.

## Results

### At inclusion

Table 1 compares the main characteristics of the patients. Beside the percentage of males averaging at 70% and age at first contact being 23 years, all remaining characteristics differ between centres, and variations are substantial. Granada, Lisbon, St Etienne and Lille have the highest percentage of young people (≤ 35 years). On average, 80% of the patients have never been married, but Dublin and Lille have slightly lower percentages. In Lisbon and Granada, close to 100% of the patients live in a private home since there are very few other resources. In the French centres, approximately 13% of the patients live in sheltered accommodation managed by the psychiatric system. Fewer patients receive social benefits in Lisbon. A minority of the patients have regular wages (11% on average).

**Table 1:** Characteristics of patient populations at entry for seven study areas in four countries

	**Lille**	**Lyon**	**La Verrière**	**St Etienne**	**Dublin**	**Lisbon**	**Granada**	**Total**	**p**
Number of patients	48	45	27	50	64	50	84	368	
Males (%)	65	73	63	78	61	82	75	70	N.S
≤ 35 years	50	36	37	57	25	56	58	46.7	0.01
Mean age at 1^st ^contact with services	20 (4.5)	21 (6.8)	24 (4.5)	22 (5.0)	26 (6.7)	23 (6.3)	23 (6.4)	23	N.S
In-patient (%)	12	27	22	28	3	0	4	12	0.00
Never married (%)	72.5	83	89	82	67	86	82	80	0.05
Sheltered accommodation* (%)	13	19	11	12	3	0	1.2	5.8	0.00
Private accommodation (%)	78	73	89	86	81	100	99	86.4	0.01
Regular wages (%)	9	10.5	11	4	12.5	16	11	11	0.00
On social benefit or pension (%)	73	76	74	83	67	24	65	65	0.00

* Homeless: 15.6% Dublin, 4 % Saint Etienne, 0% elsewhere

**Figure 1:**
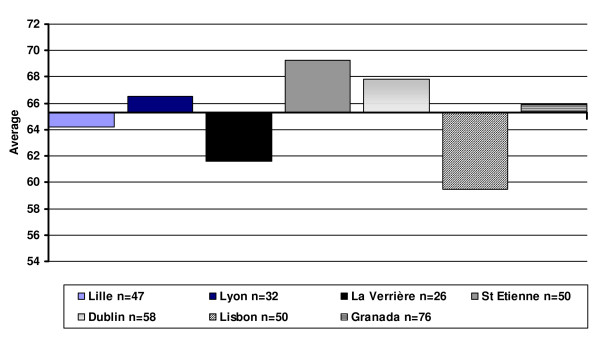
Global score, inter-centre differences at baseline.

Figure 1 presents a comparison of global QOL scores between centres at baseline using the 14-item total. It shows marked differences between centres (p = 0.002): four centres are above average and three are below.

On average, levels of satisfaction for each area vary from 5.37 for food to 3.77 for finances. Between these extremes, the areas where people are most satisfied are clothing (5.11), housing (5.03), people they live with (5.02); then come family (4.92), neighbourhood (4.83), local services (4.79), relations with others (4.70) and friends (4.59), followed by work or day schedule (4.41), spare time (4.35), health (4.31) and leisure (4.26). If the six newly added items are considered, love life is the area where people are least satisfied (3.69), whereas the one where they are most satisfied is freedom (4.90), followed by responsibilities (4.65), self-confidence (4.41), life in general (4.24) and what other people think of you (4.21).

**Figure 2:**
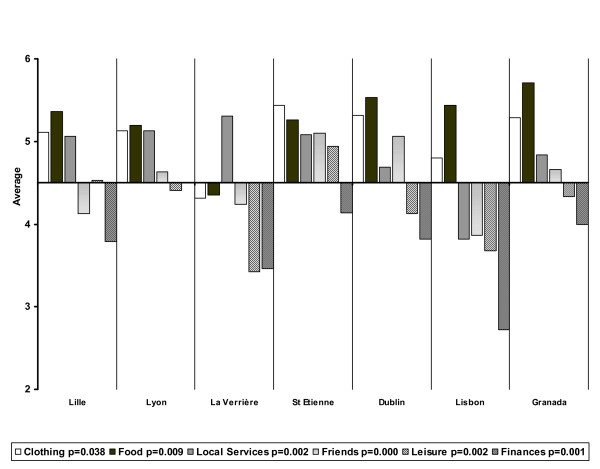
Satisfaction by area across sites.

**Figure 3:**
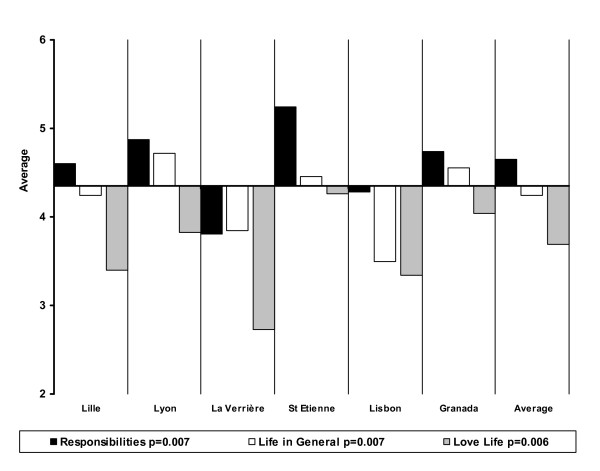
Satisfaction in additional areas across sites.

However, there are some significant differences between centres concerning many items (Figures 2 and 3). Satisfaction with finances is universally the lowest among the 14 items, but differences exist between centres (p = 0.001). Satisfaction with leisure, which is also generally low, is more contrasted, being relatively high in St Etienne and Lille and below average in La Verrière, Dublin and Lisbon (p = 0.002). Satisfaction with friends is below average in Lille, La Verrière, and Lisbon (p = 0.000). Satisfaction with love life is the lowest among the 6 additional items and this specific dissatisfaction is the highest in La Verrière, Lisbon and Lille (p = 0.006). On the other hand, satisfaction with food (p = 0.009) and clothing (p = 0.038) is the highest everywhere except in La Verrière, where people are most educated. Satisfaction with local services is especially low in Lisbon (p = 0.002). Finally, it is noteworthy that patients seem relatively satisfied with responsibilities except in La Verrière (p = 0.007).

When comparing variations of total QOL score according to the diverse sociodemographic characteristics for the entire sample at baseline, there are very few significant differences, and ones only relating to income source (p = 0.035): individuals who receive financial assistance from a partner (72.6) or their parents (68.9) are the most satisfied, followed by those who have a salary (67.8), then come those who receive unemployment benefit or social benefit (66.9), followed by those who receive disability benefits for sickness or handicap (65.7), which is the most frequent situation (129/329 cases). The persons least satisfied are those who receive their money for subsistence either from pensions (64.5), extended family or friends (60.7) and other sources of income (57.6).

**Figure 4:**
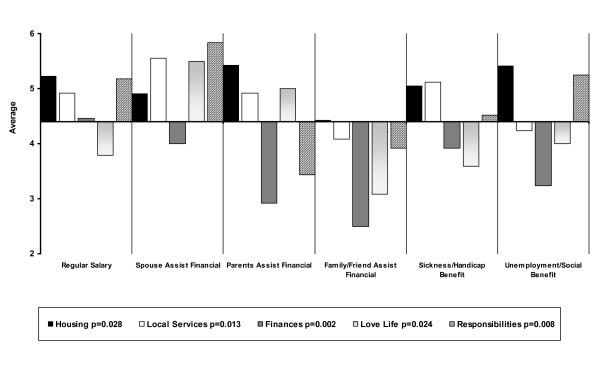
Satisfaction according to source of income.

When individual satisfaction items are compared using various sociodemographic variables few are significant. First, one of these concerns age for satisfaction with finances only (p = 0.046): the older respondents (> 35 years) are more satisfied than their younger counterparts (3.99 versus 3.56). Second, income is also linked to satisfaction in two areas: local services (p = 0.011) and finances (p = 0.008), those with the highest income being the most satisfied and those who have low income being dissatisfied. Source of income parallels this finding. Figure 4 shows that some situations present advantages and disadvantages: those who get a salary, who are helped by their partner or have a sickness or handicap benefit are the most satisfied with finances (p = 0.002) and local services (p = 0.013). People who are helped by their partner are most satisfied concerning their love life along with those who are helped by their parents (p = 0.024). However, unlike the former, the latter are least satisfied with responsibilities (p = 0.008) and finances (p = 0.002) and most satisfied with housing (p = 0.028). Those whose income is derived from unemployment benefit or social benefit are most satisfied in terms of their responsibilities just after those who are helped by their partner (p = 0.008). Third, being married is linked to better satisfaction with housing (p = 0.049) and love life (p = 0.000). Cohabitation without being married is not so good except in terms of love life, which is given poor ratings by persons divorced, separated or single. Divorced and separated persons also have lower levels of satisfaction in housing. Fourth, housing is linked to satisfaction with friends (p = 0.046) and opinion of others (p = 0.040): those who are homeless claim higher satisfaction about friendship than those living in a private house or those in psychiatric facilities either sheltered or semi-private and conversely the lowest level of satisfaction about the opinion of others than the three other groups. Finally, level of education is linked to some items: those who have had some kind of university training are the least satisfied with housing (p = 0.008), love life (p = 0.037), level of responsibilities (p = 0.002) and their life in general (p = 0.044) whereas those who have no education or a low level of education are the most satisfied in the same areas. This could explain why La Verrière, where the most educated patients are, is one of the most dissatisfied sites.

### Evolution in one year

**Figure 5:**
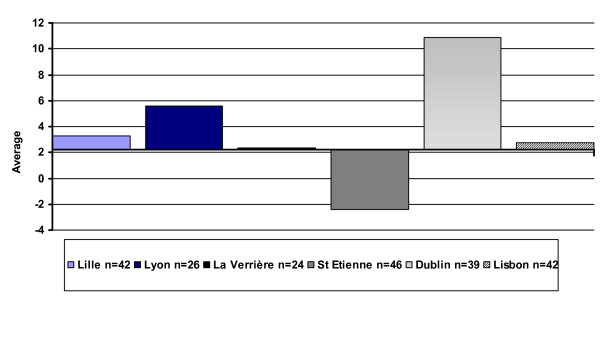
Global score, inter-centre differences between one-year follow-up and baseline.

The evolution of the global QOL scores among those respondents who took part in the follow-up (excluding the subjects from Granada) is presented in Figure 5. It is worth noting that no significant difference between one-year follow-up and baseline is found in any centre, with the exception of Dublin, which shows a significant increase (p = 0.002).

Sociodemographic and environmental variables appear to play a more important role at one-year follow-up than at baseline: with the time passing, their influence on various aspects of QOL increases.

Women's global satisfaction increases (+5.39), whereas men change only slightly (+0.92) over time (p = 0.028). Respondents who are divorced or separated (+10.8) or cohabiting (+8.28) have the highest increase (p = 0.057). Those with the lowest income progress most (+4.58) whereas, in contrast, those with the highest income decline most (-4.33; p = 0.046). Globally, the main areas subject to change over time are finances, which gains one point in each centre, and family relationship, which looses more than one point. All remaining satisfaction areas slightly increase except that of love life which decreases.

These evolutions are sometimes different by centres or according to some of the sociodemographic variables.

Satisfaction with health increases over time in all centres, except La Verrière and St Etienne where it declines (p = 0.009). Relationship with the family deteriorates over time in varying degrees (p = 0.012) in each centre (the greatest deterioration occurring in La Verrière), whereas relationship with friends improves slightly, except in Lisbon (p = 0.038), and relationship with others improves slightly, except in St Etienne (p = 0.017). Leisure improves greatly in La Verrière and in Dublin and deteriorates in St Etienne (p = 0.002).

Compared with men, women improve better their satisfaction with health (p = 0.006) and finances (p = 0.011). Respondents aged over 35 years are more satisfied at one-year follow-up with their responsibilities (p = 0.046). Income level does not appear to have a significant influence in the evolution of satisfaction except concerning local services, which is lower at one-year follow-up for everyone, excluding persons with the minimum legal wage level (p = 0.002). The same group shows also better improvement of their satisfaction concerning the opinion of others (p = 0.048) and the wealthiest improve better in terms of satisfaction about their life in general (p = 0.050). Respondents with the lowest level of education show better improvement compared with the others in terms of health satisfaction (p = 0.044). Those who live in semi-private accommodation experience increase better their satisfaction concerning their housing, unlike those living in private homes or who are homeless (p = 0.036). Satisfaction concerning relations with others change over time: the homeless improve whereas those living in sheltered accommodation deteriorate (p = 0.023). Finally, satisfaction regarding relationship with the family deteriorates, especially in the category of the divorced, separated and never-married (p = 0.000), however, satisfaction with health is better for those divorced whilst it remains unchanged for the others (p = 0.032). Global satisfaction with housing slightly increases for everyone except for those married or widowed (p = 0.038).

At baseline, no variables are found to influence the global satisfaction in a multiple regression analysis using the variables that show significant influences: centre and income level. However, when a multiple regression is conducted on the variables which have an influence on a one-year evolution of global satisfaction namely: centre, gender, income and marital status, the latter remains significant and all other influences disappear.

### Clinical variables

Concerning the global satisfaction score, patients differ across centres regarding 3 types of symptoms: slowness/under-activity, neurotic symptoms and alcohol problems (see Table 2). When the diverse relevant sociodemographic variables are entered into a variance analysis, these three clinical variables remain significant (slowness is at just p = 0.06, but neurotic symptoms are at p = 0.001 and alcohol at p = 0.004) when all remaining effects disappear, such as sex, centre, age, marital status and income level. Age/sex interaction is not significant and when country is used instead of centres the findings remain similar.

**Table 2:** Frequency of individual ongoing symptoms at inclusion (%)

	**Lille**	**Lyon**	**La Verrière**	**St Etienne**	**Dublin**	**Lisbon**	**Granada**	**p**
	N = 47*	**N = 45**	**N = 27**	**N = 50**	**N = 64**	**N = 50**	**N = 84**	
**Psychotics symptoms**	80.4	93.2	92.6	100.0	68.8	100.0	100.0	0.97
**Slowness/under-activity**	**37.0**	**65.9**	**81.5**	**68.0**	**45.3**	**62.0**	**71.6**	**.04**
**Side effects. dyskinesia**	28.3	43.2	51.9	40	26.6	48.0	27.2	0.55
**Neurotic symptoms**	**19.6**	**43.2**	**22.2**	**6.0**	**9.4**	**16.0**	**23.5**	**.009**
**Physical symptoms**	13.0	31.8	33.3	42.0	6.3	14.0	7.4	0.89
**Dangerous behaviour**	15.2	29.5	22.2	32.0	15.6	24.0	19.8	0.39
**Embarrassing behaviour**	15.2	27.3	11.1	12	9.4	40.0	24.7	0.96
**Distress**	21.7	38.6	7.4	34.0	12.5	28.0	38.5	0.60
**Alcohol**	**13.0**	**4.5**	**11.1**	**10.0**	**10.9**	**16.0**	**21.0**	**0.01**
**Drugs**	0.0	4.5	3.7	4.0	4.7	6.0	7.4	0.86

*NFCAS missing for one patient

Factors influencing differences between one-year follow-up and baseline are rather different: the only symptom which influences the global satisfaction score is dangerous behaviour (p = 0.06) and it is at a level that is only just significant.

## Discussion

Most of the differences concerning the various sociodemographic variables appeared at one-year follow-up when some patients had left and one of the centres was missing. Consequently, the differences should be interpreted with caution since the drop-out rate could be due to many reasons linked to the health status of the person. However, it is worth noting that we found no difference in baseline satisfaction between respondents and non-respondents at one-year follow-up, except in Lyon (p = 0.008) where non-respondents at one-year follow-up were associated with greater satisfaction at inclusion.

In addition, it should be noted that all patients included had to be at least once in contact with a specialised mental health care system during the year of inclusion. This excludes those who were in remission as well as those who did not have access to specialised care and unfortunately, we are not able to check any differences. Table 2, which reports their clinical needs, illustrates that at the time of evaluation these patients were highly symptomatic and few of them were homeless, reflecting a relative adequacy of the studied population.

As with the majority of QOL research in both general and mentally ill populations [30-34], we did not find any link between gender and QOL. This result could also be compared with the Vandiver [35] study on QOL, gender and schizophrenia in the United States, Canada and Cuba which reported greater QOL and satisfaction with social relationships for females in Canada, the reverse with the Cuban sample and no difference between genders in the United States. However, our study shows differences by sex in evolution over one year as in the study of individuals with severe mental illness, conducted by Roder-Wanner, Oliver and Priebe [36], which found that QOL predictors differed according to gender and gave support to the existence of gender-specific processes and contexts of subjective evaluation.

We did not find many differences between young and old patients, but we did find one concerning finances, which was in favour of the older respondents as in the case with most QOL studies done among the general population and the mentally ill, which found that the older the population the more satisfied they are with their QOL, particularly in the area of finances [33,34,37].

Living conditions, such as living arrangements, have been found to impact on the subjective QOL of individuals with severe mental illness. In all studies, the least restrictive living arrangements were associated with better QOL [34,38,39]. In our study, we did not find many differences except in relations with friends and the perception of the opinion of others, but we failed to find any difference in satisfaction regarding freedom or responsibilities.

A study by Fabian [40] showed that, while there was no significant difference between the working and non-working groups on the basis of work status alone, gender appeared to mediate the relationship between employment and QOL indicators. As predicted, working males were the most satisfied group and non-working males the least satisfied. However, working females expressed less satisfaction across all subjective life domains studied than did non-working females. In our study, those who declared they were working were not more satisfied than those persons who were helped by their spouse or parents, but they were more satisfied than pensioners or people living on disability benefits.

Most of the epidemiological research to date has systematically found increased well-being and mental health in people with higher levels of education [35,41]. However, we found completely inverse results, which fit in with C. Mercier's theory about satisfaction and expectation in which the more educated have higher aspirations leading to a greater distance between where they are and where they would like to be and resulting in their feeling more dissatisfied than people with a lower level of education [42].

Global comparisons by centres may also reflect the global resources provided to those suffering from severe mental health disorders. In a similar attempt to compare diverse centres in diverse countries, Gaite et al [43] state that contrary to what has been claimed QOL measures reflect more subjective measures than environmental conditions. Some of their findings are in favour of an accurate relation to environment. In our data, the Lisbon patients' dissatisfaction with finances reflects their environmental situation as well as their high dissatisfaction with services, leisure and friends since most of them were living within their families with no outside resources at all.

Finally, according to the evolution over a one-year period, marital status appears to be one of the most important variables in terms of satisfaction in many areas, as shown in the work of Salokangas and colleagues [44], which found greater satisfaction in its follow-up among women and married men. The results of Salokangas's studies strongly emphasise that relations between gender, marital status and QOL to a great extent depend on the study sample and may vary by study area.

## Conclusion

This study replicates many previous findings, however, its originality stems from the variety of EU countries where the patients were treated: these countries have quite different resources, medical as well as social, and the living conditions of patients are very different. However, the main differences as far as their QOL is concerned depend very much on extra-psychiatric variables, principally marital status and income and not so much on clinical variables.

Concerns of patients are very similar to those of the non patient populations and income is a serious concern: finances and relationships with others, including love life, are the areas where they are the most dissatisfied.

QOL integrates many dimensions and this work shows that an improvement in an area may raise problems in another: for example, being in sheltered housing increases satisfaction with housing but at the same time seems to deteriorate the relationship with others while making no difference in remaining areas.

Globally, with largely different resources, after one year spent under the diverse psychiatric care systems, the patients' satisfaction with finances increases, which may indicate some efficiency on the part of psychiatric teams in gathering subsidies for them. However, satisfying relationships with others including love life are a far more difficult goal to reach for such patients with chronic schizophrenia who experience considerable handicaps in those areas essential for their own conception of QOL.

## Abbreviations

QOL Quality of Life

ICD International Classification of Diseases

SCAN Schedules for Clinical Assessment in Neuropsychiatry

SLDS Satisfaction with Life Domains Scale

PHSD Past History and Sociodemographic Description schedule

NFCAS Needs For Care Assessment Schedule

## Competing interests

The author(s) declare that they have no competing interests.

## Authors' contributions

VKM was involved in the design of the study, statistical analysis and drafting of the manuscript. MX, BMK, JD, EL, JP, JLR and DW participated in the design of the study and data collection, and provided comment on the content of the manuscript. AS and CSD were involved in the revision of the manuscript.

## Pre-publication history

The pre-publication history for this paper can be accessed here:


